# Extraction of *Radix trichosanthis* Polysaccharides for Potential Antihyperlipidemic Application

**DOI:** 10.1155/2022/3811036

**Published:** 2022-04-11

**Authors:** Fujia Chen, Yilin Xu, Nana Ding, Hongyan Li, Tongbiao Li, Fengyun Liu, Mengxue Liang, Li Song, Junhe Liu, Enzhong Li, Jiayang Liu

**Affiliations:** ^1^College of Biology and Food Engineering, Huanghuai University, Zhumadian 463000, China; ^2^College of Plant Protection, Henan Agricultural University, Zhengzhou 450046, China; ^3^School of Environmental Science and Engineering, Nanjing Tech University, Nanjing 211816, China

## Abstract

This study focused on the optimization of ultrasound-assisted compound enzyme extraction for polysaccharides (RTPs) from *Radix trichosanthis* by orthogonal experiment and response surface methodology, and then its extraction kinetics model and antihyperlipidemic activities were studied. The optimum extraction process was as follows: cellulase—1.0%, papain—1.0%, pectase—0.5%, pH—5, extraction temperature—50°C, and liquid-to-solid ratio—30 mL/g; prediction value of RTPs was 7.54%; the experimental yield of RTPs was 7.22%, while 50 minutes was optimized in Weibull kinetics model. Then high-dose groups of RTP extract could reduce the TC, TG, and LDL-C levels and increase the level of HDL-C in high-fat mice, with the ability to lower the MDA content and enhance SOD level.

## 1. Introduction

*Radix trichosanthis*, also known as snake gourd root, is the dried root of *Trichosanthes kirilowii* Maxim, which is a perennial vine of Cucurbitaceae family. As a well-known Chinese remedy for diabetes, *Radix trichosanthis* was originally referenced in Shennong Bencao Jing, the earliest therapeutic medicine in China. Its potential application was widely described in promoting fluid relieving thirst, clearing heat and fire, swelling, and evacuating pus [1]. Currently, assorted proteins and peptides reported so far were mainly concentrated in *R. trichosanthis*. Several had been isolated that exhibited cytotoxicity to tumor cells and hypoglycemic effects. Other active compounds in *R. trichosanthis* included nucleosides, amino acids, and saccharides, polysaccharides, and saponins [2]. The polysaccharides of *R. trichosanthis* (RTPs), the main active ingredient in *R. trichosanthis*, have an immunoregulatory function and antitumor activity [3]. Antilipidemic activities of the RTPs extract have not previously been reported as far as we know. Hyperlipidemia is a common metabolic syndrome worldwide that mainly manifests as an abnormal increase in serum lipid and lipoprotein content. An unhealthy lifestyle and diet might be considered as one of the reasons for hyperlipidemia as a major complication of obesity [4, 5].

Polysaccharides, a group of biological macromolecules, have been extensively studied regarding their antioxidation, immunoregulation, and antihyperlipidemic activities [6]. Extraction methods can not only significantly affect the content and yield of polysaccharides but also affect the structural characteristics and biological activities of polysaccharides [7]. Ultrasonic extraction can destroy cell walls and improve mass transfer and penetration effectively [8]. Enzymatic extraction can obtain target compounds at a lower cost with less degradation simultaneously [6]. Hence, ultrasound-assisted compound enzymatic extraction (UACEE) utilizes the synergistic effect of enzymatic hydrolysis and ultrasound, which can meet the industry for the preliminary extraction of polysaccharides [9].

Therefore, this study is aimed at optimizing extraction of RTPs by response surface methodology (RSM) and at studying the interactions between independent variables and UACEE. Furthermore, the kinetic model and the antihyperlipidemic activity of RTPs were evaluated in hyperlipidemic mice induced by a high-fat diet (HFD).

## 2. Materials and Methods

### 2.1. Chemicals and Reagents

*R. trichosanthis* and Simvastatin were acquired from a local pharmacy. Glucose standard was purchased from Sigma (St. Louis, MO, USA). Cellulase (20 U/mg) [3], papain (20 U/mg), and pectase (25 U/mg) were obtained from Jinsui Biotechnology Co., Ltd. (Shanghai, China). Detection kits for the total cholesterol (TC), triglyceride (TG), high-density lipoprotein cholesterol (HDL-C), and low-density lipoprotein cholesterol (LDL-C), superoxide dismutase (SOD), and Maleic Dialdehyde (MDA) were procured from Nanjing Jiancheng Biology Engineering Institute. All other analytical-grade chemicals were obtained from Nanjing Reagent Co. Ltd. (Nanjing, China).

### 2.2. Extraction of RTPs

*R. trichosanthis* powders were extracted in an ultrasonic cell disintegrator (JY92-2D, Ningbo Scientz Biological Technology Co., Ltd., Ningbo, China). The crude RTP yield (%) was analyzed by a phenol-sulfuric acid method as our previous studies [6] and calculated as follows:

(1) $$\begin{array}{c} \text{Yield}\left(\%\right)=\frac{C\times V}{W}. \end{array}$$

*C* (mg/L) is the concentration of polysaccharides, *V* (mL) is the volume of solution, and *W* (g) is the weight of the dried sample.

### 2.3. Single Enzyme Screening and Orthogonal Test of the Proportion of Compound Enzymes

Firstly, the extractions were carried out with cellulase, papain, and pectase of different substrate concentrations (0.5%, 1.0%, 1.5%, 2.0%, and 2.5%), respectively. The other conditions were fixed as follows: ultrasonic power of 200 W, pH value of 5.5, extraction temperature of 50°C, extraction time of 50 min, and the liquid-to-solid ratio of 30 mL/g. Secondly, an orthogonal *L*_9_ (3^4^) test design was used to optimize the proportion of compound enzymes on the basis of the single-factor test, while the other experimental factors were kept constant as a single-factor test. Then, the best proportion of compound enzymes was selected for subsequent experiments.

### 2.4. Single Factor of UACEE

Based on the enzyme screening test, the effects of extraction time, pH, extraction temperature, and liquid-to-solid ratio were studied for the single factor of UACEE as follows: one experimental factor was changed while the others were kept constant [6].

### 2.5. RSM for UACEE

On the basis of orthogonal test of the proportion of compound enzymes and the single-factor of UACEE, a Box-Behnken design (BBD) with three variables (*X*_1_, pH; *X*_2_, extraction temperature; *X*_3_, liquid-to-solid ratio) at three levels was used to determine further the optimum UACEE condition of RTPs extraction [7]. All 17 experiments were performed at random to minimize systematic errors. Multiple regressions analyzed data from BBD to fit the following quadratic polynomial model:

(2) $$\begin{array}{c} Y=\beta_{0}+\overset{3}{\underset{i=1}{\sum }}\beta_{i}X_{i}+\overset{3}{\underset{i=1}{\sum }}\beta_{ii}{X_{i}}^{2}+\overset{3}{\underset{i=1}{\sum }}\overset{3}{\underset{j=i+1}{\sum }}\beta_{ij}X_{i}X_{j}, \end{array}$$

where *Y* is the predicted response, *X*_*i*_ and *X*_*j*_ are the independent variables, and *β*_*i*_, *β*_0_, *β*_*ii*_, and *β*_*ij*_ represent the coefficients of the linear, constant, quadratic, and interaction, respectively. *β*_*i*_ is the main effect. Design-Expert software (version 8.0.6.1, State-East, Inc., Minneapolis, USA) was utilized for the experimental design, data analysis, and model building. According to the one-way analysis of variance (ANOVA), the effect and regression coefficients were measured, and three-dimensional (3D) surface and contour plots were generated.

### 2.6. Kinetic Analysis

Based on the optimum extraction conditions of UACEE by RSM, a classical kinetic model was used to analyze the extraction process; the single factor experiment of extraction time (0-60 min) was carried out. The experimental data fit well to the Weibull model, which was so simple that it was widely used in extracting polysaccharides from biomaterials [10]. Using Origin 9 (Microcal Origin, USA), the stable yield of *R. trichosanthis* polysaccharide ([RTPs]_∞_) and extraction rate constant (*k*_*e*_) were obtained from the nonlinear regression.

(3) $$\begin{array}{c} \left[\text{RTPs}\right]\left(\%\right)={\left[\text{RTPs}\right]}_{\infty }\left(1-e^{-{\left(k_{e}t\right)}^{d}}\right), \end{array}$$

where *d* represents shape parameter. When *d* = 1, Weibull is reduced to a first-order equation, and the whole curve is a satisfactorily fit to an exponential equation [11].

### 2.7. Animals and Antihyperlipidemic Activities

Lyophilized RTPs were obtained under optimal conditions according to our previous research for further antihyperlipidemic activity assays [6]. A total of 50 male Kunming strain mice (specific pathogen-free (SPF), 20 ± 2 g) were purchased from the animal center of Henan province, China [SCXK (Yu) 2017-0001]. The animal cares and studies were approved by the Ethics Committee of Huanghuai University (Zhumadian, China), which insisted on strict adherence to the criteria outlined in the “Guide for the Care and Use of Laboratory Animals” prepared by the National Academy of Sciences and published by the National Institutes of Health [12]. The mice were housed in cages in standard environmental conditions (25 ± 2°C, 50-70% relative humidity, 12 h light-dark cycle) and allowed for access to water and food at will. After being acclimatized for 7 days, the mice were randomly divided into 5 groups, including the normal control group [13], hyperlipidemic control (HC), positive control (PC), high-dose group (HD), and low-dose group. Group NC mice were fed a standard diet purchased from the animal center of Henan province in China, while the other four groups were fed a HFD obtained from Botai Hongda Biotechnology Co., Ltd. (Beijing, China) for 60 consecutive days to establish the hyperlipidemia model successfully by testing the serum lipid levels. Group PC mice were fed HFD and treated with 5 mg/kg Simvastatin daily. Group HD and LD mice were fed HFD and treated with 300 mg/kg and 100 mg/kg RTPs per day, respectively. All the treatments were intragastric administrations [14] for 28 days. The mouse was weighed every 7 days. At the end, food was withheld from mice for at least 12 h, and blood samples were collected from the eyeballs in 2 mL heparinized tubes. The organs (liver, spleen, and kidney) were also collected for calculating the relative organ weight (%) and the following work. The serum was separated by centrifugation at 3500 rpm at 4°C for 15 min, and serum concentrations of TC, TG, LDL-C, HDL-C, SOD, and MDA were measured.

(4) $$\begin{array}{c} \text{Relative organ weight }\left(\%\right)=\frac{\text{weight of mice organ }\left(\text{g}\right)}{\text{body weight of the final experimental day }\left(\text{g}\right)\;}\times 100. \end{array}$$

### 2.8. Statistical Analysis

All trials were carried out in duplicate (3 or 6 repeats), and the data were presented as the means ± standard deviation (SD). Statistical analysis was carried out by one-way analysis of variance (ANOVA) by SPSS software version 20.0 (SPSS Inc., Chicago, IL, USA).

## 3. Results and Discussion

### 3.1. Effects of Enzymes on RTP Yield

**Figure 1:**
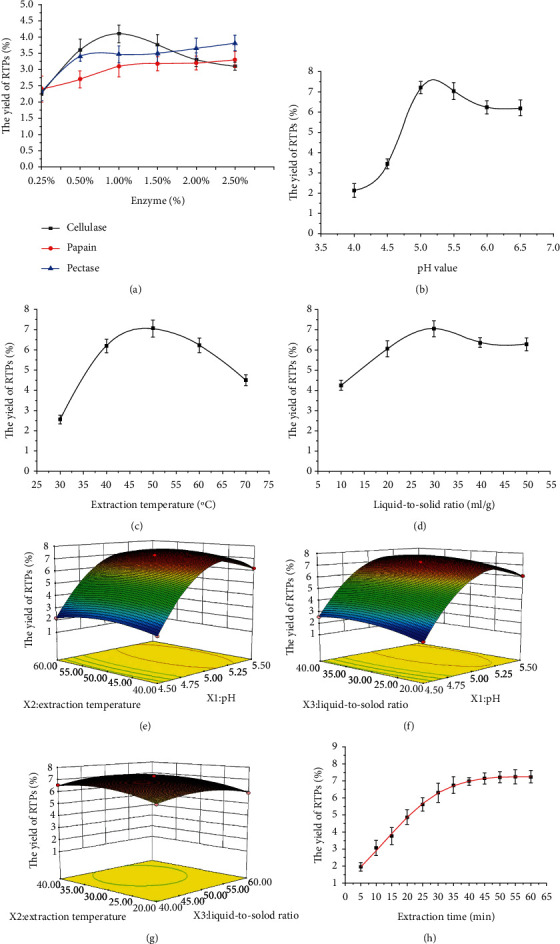
Effect on the extraction yield of RTPs. (a) A single factor for different concentrations of enzymes, (b) single factor for pH, (c) single factor for extraction temperature, and (d) single factor for liquid-to-solid ratio. (e) Response surface (3D) plot of interaction between pH (*X*_1_) and extraction temperature (*X*_2_), (f) response surface (3D) plot of interaction between pH and liquid-to-solid ratio (*X*_3_), and (g) response surface (3D) plot of interaction between extraction temperature and liquid-to-solid ratio. (h) Kinetics of UACEE process described by Weibull model. Notes: [RTPs]∞ = 7.8374%; *R*^2^ = 0.9936; *k*_*e*_ = 0.0521 min^−1^; *d* = 0.9825.

The effects of different enzymes with different concentrations were investigated in UACEE and shown in Figure 1(a). The maximum yield of RTPs was 4.1 ± 0.27% when cellulase concentration was 1.0%, so the optimum concentration of cellulase was 1.0%. Meanwhile, the yield of RTPs increased slightly when the concentration of papain was over 1.0%, so the optimum concentration of papain was 1.0%. When pectase concentration was 0.5%, the yield of RTPs increased rapidly up to 3.4 ± 0.17%. Then, with the increase of pectase concentration, the yield of RTPs increased slowly. From an economical point of view, 0.5% was chosen as the suitable concentration of pectase.

### 3.2. Orthogonal Result

An orthogonal test with three factors and three levels was designed to analyze the optimal proportions of enzymes (Table 1). From the range analysis, the yield of RTPs was increased from 2.76% to 6.89%; the significance order of these three factors was *A* > *B* > *C* (cellulase > papain > pectase). Cellulase played an important role in the yield of RTPs during the UACEE process, because cellulase might degrade cell wall efficiently and separate polysaccharides from cell tissues sufficiently and quickly. The optimum was *A*_2_*B*_2_*C*_1_ (cellulase (1.0%), papain (1.0%), and pectase (0.5%)), while the verified experimental yield was 6.96%, and this condition was selected for subsequent experiments. Subsequently, ANOVA in an orthogonal test indicated that cellulase was the most significant factor (*p* < 0.05, Supplementary Table S1).

### 3.3. Single-Factor Experiment of UACEE

Based on the best proportion of the compound enzymes, the effects of a single factor of UACEE on the extraction yield of RTPs were depicted in Figures 1(b)–1(d). When pH fixed at 5.5, liquid-to-solid of 30 mL/g, extraction temperature was 50°C at 20, 30, 40, 50, and 60 min; the yields of RTPs were 3.26, 5.22, 6.81, 7.05, and 7.01, respectively. So 50 min was the selective extraction time (Fig. S1) in the following work.

The extraction process of UACEE was performed for 50 min, fixed ultrasonic power (200 W), a fixed proportion of the compound enzymes to investigate different pH values (4.0 to 6.5), extraction temperatures (30 to 70°C), and liquid-to-solid ratio (10 to 50 mL/g), respectively. The yield of RTPs increased with pH increasing with a peak value of 7.22% at pH 5.0 (Figure 1(b)), with a fixed extraction temperature of 50°C and a liquid-to-solid ratio of 30 mL/g. Further increase of pH led to a decrease in the yield of RTPs, which might be ascribed to poor enzyme activities with the changes of their conformations at unsuitable pH values [15]. Therefore, 5.0 was the right pH value.

Then pH of 5.5 and liquid-to-solid ratio of 30 mL/g were fixed.  Figure 1(c) reveals that the extraction yield rose from 2.56% to 7.05% with the extraction temperature increasing and peaked at 50°C. Further increase of temperature did not enhance the RTPs yield, which was in good agreement with the previous reports [16]. Thus, 50°C was the most appropriate extraction temperature in the present study.

Screening of a proper liquid-to-solid ratio [17] could facilitate the movement of the plant cells to the active sites of the enzymes and the dissolubility of RTPs in solvent [18]. The effect of different liquid-to-solid ratios on the yield of RTPs was shown in Figure 1(d), with a fixed pH of 5.5 and an extraction temperature of 50°C. The yield of RTPs increased markedly with a peak value of 7.05% at 30 mL/g. Therefore, 30 mL/g was selected as the optimum ratio of liquid-to-solid for the following work.

### 3.4. RSM and Analysis

BBD experiment was performed with three independent variables (*X*_1_, pH value; *X*_2_, extraction temperature; *X*_3_, liquid-to-solid ratio) (Table 2). An equation was obtained to describe the causal relationship between predicted response variable on RTP yield (*Y*) and three independent variables:

(5) $$\begin{array}{c} \text{Yield}=-286.35925+105.6375X_{1}+0.51162X_{2}+0.40715X_{3}-5.0\times 10^{-4}X_{1}X_{2}-0.019\;X_{1}X_{3}+1.0\times 10^{-4}\;X_{2}X_{3}-10.101X_{1}^{2}-5.1025\times 10^{-3}\;X_{2}^{2}-4.7775\times 10^{-3}\;X_{3}^{2}. \end{array}$$

It indicated that the equation was of high significance considering the high *F* value (*F*_model_ = 1518.86) and low *p* value (*p*_model_ < 0.0001, Supplementary Table S2. *F* value (5.59) and *p* value (0.0649) of lack-of-fit not only implied that it was insignificant relative to the pure error due to noise but also confirmed the validity of the model [6]. *R*^2^ was close to 1.0, andcoefficient variation value < 5%(C.V.% = 1.23%), which indicated the model was reliable, precise, and adequate for prediction within the range of these variables [16, 17]. The variables with the largest influence were *X*_1_ (pH), *X*_3_ (liquid-to-solid), and the quadratic coefficient *X*_1_^2^, *X*_2_^2^, *X*_3_^2^ (*p* < 0.01), while the interaction coefficient *X*_1_*X*_3_ of pH and liquid-to-solid had significant effects (*p* < 0.05); the other coefficients had no significant effect on the yield of RTPs (*p* > 0.05). The interactions between the variables, the relationship between responses, and experiment levels were depicted and visualized by 3D response surface plot (Figures 1(e)–1(g)). At the bottom of 3D surface plots, 2D contour plots with two tested variables indicated the interactions between the variables. An elliptical 2D contour plot meant that the interactions between the corresponding variables were significant, while a circular contour plot indicates otherwise [19]. The variance analysis showed that the interaction between pH (*X*_1_) and liquid-to-solid ratio (*X*_3_) is significant. As shown, the optimal extraction conditions for RTPs extraction were predicted as follows: pH—5.17, extraction temperature—46.54°C, and liquid-to-solid ratio—29.90 mL/g. Under optimal conditions, the maximum predicted yield was 7.54%. Actually, the veritable condition might be recommended as follows: pH value—5.0, extraction temperature—50°C, and liquid-to-solid ratio—30 mL/g, with the experimental yield of 7.22%, which was very close to the predicted yield (C.V.% = 4.24%, <5%).

### 3.5. Kinetic Analysis

With distilled water, UACEE extraction was solid-liquid extraction [10, 11]. In UAEE systems, kinetic processes were established, and extraction time was also optimized. The mass transfer process of RTPs and detailed extraction indicated that a good fit of the model with *R*^2^ of 0.9936 (Figure 1(h)). Other model parameters were as follows: [RTPs]_∞_—7.8374%; *k*_*e*_—0.0521 min^−1^; *d*—0.9825. The simulated curve was shaped as previous research for extracting various bioactive components from plant materials [9, 11]. There were two extraction stages clearly visible: (a) the rapid stage appeared within the first 30 minutes. At this stage, 80.6% RTPs were transferred to the solvent quickly at a constant speed. (b) Slow stage occurred slowly for further extraction progresses and lasts about 30 minutes. With slow diffusion of RTPs at this stage, it was probably due to the depletion of the finite RTPs. Therefore, the optimal extraction time of RTPs was 50 min, which was close to getting the stable yield of RTPs. With the increase of time, the contents of polysaccharides might increase, while the activity and the structure of the polysaccharide might be destroyed [19].

### 3.6. Antihyperlipidemic Effect

**Figure 2:**
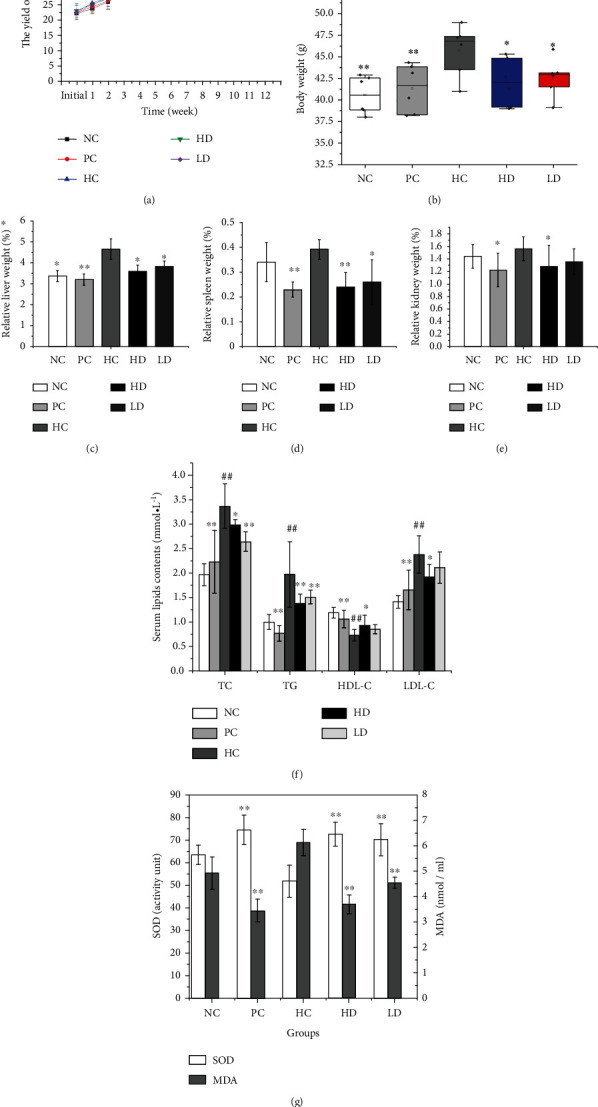
The indexes of antihyperlipidemic activities in high-fat mice. (a) Mouse body weights in different groups from the initial to 12 weeks. (b) Boxplots of body weights in various groups at the end of the experiment. (c–e) The relative liver, spleen, and kidney weights of variety of mice, respectively. (f) The serum lipid levels of high-fat mice treated with RTPs. (g) Serum SOD activity levels and MDA content in different groups (*n* = 6). NC: normal control group; HC: hyperlipidemic control; PC: positive control; HD: high-dose group; LD: low-dose group. Data expressed as the mean ± SD. ^∗∗^*p* < 0.01 and ^∗^*p* < 0.05 compared with the HC group; ^##^*p* < 0.01 compared with the NC group.

During the procedure, the body weights were recorded every week (Figure 2(a)). After 60 consecutive days (8 weeks) of HFD feeding, the body weights of the PC, HC, HD, and LD groups were higher than those of the NC group. Meantime, the serum TC, TG, and LDL-C levels in groups with HFD feeding increased vastly compared to NC mice, and it suggested that the hyperlipidemia model was successfully established.

After intragastric administrations for 28 days (9-12 weeks), the body weights of different groups are exhibited in Figure 2(b). From the boxplot of the body weights in various mice, the NC, PC, HD and LD groups were significantly lower than the HC group (*p* < 0.05). Particularly in the NC and PC groups, there were very significant differences from the HC group (*p* < 0.01). The relative liver weights in the NC, PC, HD, and LD groups were significantly lower than those in HC mice (*p* < 0.05), especially in PC mice, the relative liver weight was very significantly lower than the HC group (Figure 2(c)). The relative spleen weights in the PC, HD, and LD groups were significantly lower than HC mice (*p* < 0.05); especially in PC and HD mice, the relative spleen weights were very significantly lower than the HC group (Figure 2(d), *p* < 0.01). However, there was no significant difference between the NC and HC groups. The relative kidney weights of different groups in Figure 2(e) indicated that there were significant difference in PC and HD mice from the HC group, but not in NC and LD mice compared to the HC group. The serum TC, TG, and LDL-C levels in HC group mice were significantly higher than those in NC group mice (*p* < 0.01), while the serum HDL-C level in HC group mice decreased dramatically compared with that in NC group mice (*p* < 0.01, Figure 2(f)). The serum TC, LDL-C, and TG levels decreased in HD and PC group mice, compared with that of HC group. The difference was obvious. The serum TC and TG levels in LD group mice were reduced dramatically compared with HC group (*p* < 0.01), except for serum LDL-C level. It was indicated that RTPs had antihyperlipidemic effects. With the increasing of the dosages of RTPs, the activities might increase. Simvastatin exerted more pronounced antihyperlipidemic effects than RTPs.

The level of SOD in the PC, HD, and LD groups was remarkably higher than that in the HC group and NC group, but the MDA accumulation increased apparently in the HC group; the MDA level in the PC, HD, and LD groups was significantly (*p* < 0.01, Figure 2(g)) lower than that in the HC group.

## 4. Discussion

At present, the industrial production and large-scale application of RTPs were limited by traditional methods such as water bath extraction with numerous disadvantages of lower efficiency, etc. [6, 16]. Meanwhile, the extraction of plant polysaccharides by UACEE could significantly shorten the extraction time and temperature, improve extraction efficiency, and also effectively reduce the degradation of polysaccharides and loss of polysaccharide activity [19]. Referring to reports on the extraction of polysaccharides by complex enzymes [7, 9, 15, 19, 20], we obtained the appropriate ratio of complex enzymes through orthogonal experiments and then applied RSM to optimize other extraction conditions. So far, the orthogonal test had been widely applied to optimize the process, the representative factors were selected, and the first order effects of factors were fully understood [21]. It was simple and easy to implement but could not be comprehensive enough to investigate the interaction of each factor [19]. RSM is another empirical modeling technique that allowed simultaneous optimization of multiple factors on a larger scale with fewer test units than traditional designs [22]. It not only saved lots of times and resources but also accounted for the interaction of experimental factors. Especially, the BDD method, a type of RSM, was more efficient; its experiments were easier to organize and interpret [15]. In subsequent works, further optimization including the dosages of various enzymes in RSM would require investigation.

Obesity is associated with several diseases, such as diabetes, hypertension, and hyperlipidemia [23]. The commercial clinically hypolipidemic drugs Simvastatin often cause undesirable adverse effects such as rhabdomyolysis [23]. Therefore, many Chinese herbal medicines have antihyperlipidemic effects [24, 25], and the possible effective components might be polysaccharides. In our research, the antihyperlipidemic activities of *Radix trichosanthis* polysaccharides were explored. It was found that the high- and low-dose groups of RTP extract could significantly reduce TC and TG levels. It was consistent with previous reports [3]. The high-dose group of RTPs could increase the content of HDL-C and significantly reduce the content of LDL-C in mice. As well known, serum TC, TG, LDL-C, and HDL-C levels are key indicators for evaluating lipid metabolism in hyperlipidemia model caused by obesity [26]. Due to abnormal adipocyte differentiation and the imbalance of lipogenesis and lipolysis [27]. With the increase of serum TG, TC, and LDL-C levels, the incidence of cardiovascular diseases would increase. In addition, oxidative stress was closely related to the occurrence of hyperlipidemia, obesity, etc. With a long-term high-fat diet, the imbalance of free radicals might be caused in the body, which produced substantial free radicals and resulted in oxidative stress [28]. Consequently, the level of lipid oxidation was increased in hyperlipidemia mice with high-fat diet, which caused by oxidative damage. It was found that the antioxidative effect of RTPs, with the ability to lower the MDA content and enhance SOD level in high-dose group mice. These results further supported the idea that the administration of RTPs could intervene on oxidative damage caused by high-fat diet. As reported, *Radix trichosanthis* might increase in the risk of clinical adverse reactions such as skin rash and anaphylactic shock [3], when it was combined and used with some traditional Chinese medicine such as Chuanwu, Fuzi, or Caowu, but there were no evidences for the toxicity of RTPs. For the future improvement, the other assays of RTPs would be performed such as the toxicity studies and the other bioassay activities. And more tests would be done to prove the ability of lipid antiperoxidation of RTPs, such as GSH-Px (glutathione peroxidase); then the mechanism might be explored at the molecular level.

## 5. Conclusion

The extraction process of RTPs by UACEE was optimized by the single factor method and response surface methodology. The optimum extraction conditions of RTPs by UACEE are as follows: cellulose—1.0%, papain—1.0%, pectase—0.5%, pH value—5, extraction temperature —50°C, and the liquid-to-solid ratio—30 mL/g, and the experimental yield of RTPs was 7.22%, which was not significantly different from the theoretically predicted value. Weibull was used in the kinetic model, and the optimal extraction time was 50 minutes. Then, the antihyperlipidemic activities of RTPs were explored. It was found that the high-dose groups of RTPs extract could reduce the total cholesterol (TC), triglycerides (TG), and low-density lipoprotein cholesterol (LDL-C) levels and increase the level of high-density lipoprotein cholesterol (HDL-C) in mice, with the ability to lower the MDA content and enhance SOD level. This study showed that RTP extract had a certain effect in reducing hyperlipidemia.

## Figures and Tables

**Table 1:** L_9_ (3^4^) orthogonal experimental design and results.

No.	Factor			The yield of RTPs (%)
	*A*: cellulase	*B*: papain	*C*: pectase	
1	1 (0.5%)	1 (0.5%)	1 (0.5%)	2.76
2	1 (0.5%)	2 (1.0%)	2 (1.0%)	3.42
3	1 (0.5%)	3 (1.5%)	3 (1.5%)	2.85
4	2 (1.0%)	1 (0.5%)	2 (1.0%)	4.36
5	2 (1.0%)	2 (1.0%)	3 (1.5%)	6.89
6	2 (1.0%)	3 (1.5%)	1 (0.5%)	6.16
7	3 (1.5%)	1 (0.5%)	3 (1.5%)	4.12
8	3 (1.5%)	2 (1.0%)	1 (0.5%)	6.45
9	3 (1.5%)	3 (1.5%)	2 (1.0%)	5.8
K_1-1_	3.01	3.747	5.123	
K_1-2_	5.803	5.587	4.527	
K_1-3_	5.457	4.937	4.62	
R	2.793	1.84	0.596	
Optimum	*A*_2_	*B*_2_	*C*_1_	

**Table 2:** Observed responses of Box–Behnken design for the yield of RTPs.

No.	*X*_1_ (pH)	*X*_2_ (extraction temperature, °C)	*X*_3_ (liquid-to-solid ratio, mL/g)	*Y* (yield of RTPs, %)
1	4.50	40.00	30.00	2.13
2	5.50	40.00	30.00	6.2
3	4.50	60.00	30.00	2.17
4	5.50	60.00	30.00	6.23
5	4.50	50.00	20.00	1.87
6	5.50	50.00	20.00	6.06
7	4.50	50.00	40.00	2.56
8	5.50	50.00	40.00	6.37
9	5.00	40.00	20.00	5.86
10	5.00	60.00	20.00	5.88
11	5.00	40.00	40.00	6.56
12	5.00	60.00	40.00	6.62
13	5.00	50.00	30.00	7.19
14	5.00	50.00	30.00	7.17
15	5.00	50.00	30.00	7.21
16	5.00	50.00	30.00	7.24
17	5.00	50.00	30.00	7.27
Optimum	5.17	46.54	29.90	7.54

## Data Availability

No data were used.

## Supplementary Materials

Supplementary Materials Fig. S1: effect of extraction time on the extraction yield of RTPs (pH = 5.5, liquid-to-solid ratio = 30, extraction temperature = 50°C). Supplementary Table S1: one-way analysis of variance (ANOVA) in an orthogonal test. Supplementary Table S2: estimated regression coefficients for the quadratic polynomial model and the analysis of variance for the experimental results

## References

[1] Kang C., Lv C., Yang J. (2020). A practical protocol for a comprehensive evaluation of sulfur fumigation of Trichosanthis radix based on both non-targeted and widely targeted metabolomics. *Frontiers in Plant Science*.

[2] Zhang H. Q., Liu P., Duan J. A. (2019). Comparative analysis of carbohydrates, nucleosides and amino acids in different parts of Trichosanthes kirilowii maxim. By (ultra) high-performance liquid chromatography coupled with tandem mass spectrometry and evaporative light scattering detector methods. *Molecules*.

[3] Yu X., Tang L., Wu H. (2018). Trichosanthis Fructus: botany, traditional uses, phytochemistry and pharmacology. *Journal of Ethnopharmacology*.

[4] Yuan Y., Liu Q., Zhao F., Cao J., Shen X., Li C. (2019). Holothuria leucospilota polysaccharides ameliorate hyperlipidemia in high-fat diet-induced rats via short-chain fatty acids production and lipid metabolism regulation. *International Journal of Molecular Sciences*.

[5] Liu Y., Sun J., Rao S., Su Y., Yang Y. (2013). Antihyperglycemic, antihyperlipidemic and antioxidant activities of polysaccharides from *Catathelasma ventricosum* in streptozotocin-induced diabetic mice. *Food and Chemical Toxicology*.

[6] Chen F., Li D., Shen H. (2017). Polysaccharides from *Trichosanthes Fructus* via ultrasound-assisted enzymatic extraction using response surface methodology. *BioMed Research International*.

[7] Chen R., Li S., Liu C., Yang S., Li X. (2012). Ultrasound complex enzymes assisted extraction and biochemical activities of polysaccharides from *Epimedium* leaves. *Process Biochemistry*.

[8] Sajjadi B., Asgharzadehahmadi S., Asaithambi P., Raman A. A., Parthasarathy R. (2017). Investigation of mass transfer intensification under power ultrasound irradiation using 3D computational simulation: a comparative analysis. *Ultrasonics Sonochemistry*.

[9] Wu H., Zhu J., Diao W., Wang C. (2014). Ultrasound-assisted enzymatic extraction and antioxidant activity of polysaccharides from pumpkin (*Cucurbita moschata*). *Carbohydrate Polymers*.

[10] Cheung Y.-C., Siu K.-C., Wu J.-Y. (2013). Kinetic models for ultrasound-assisted extraction of water-soluble components and polysaccharides from medicinal fungi. *Food and Bioprocess Technology*.

[11] Kitanović S., Milenović D., Veljković V. B. (2008). Empirical kinetic models for the resinoid extraction from aerial parts of St. John's wort (*Hypericum perforatum* L.). *Biochemical Engineering Journal.*.

[12] National Research Council (1996). *Guide for the Care and Use of Laboratory Animals*.

[13] Chaiwong S., Chatturong U., Chanasong R. (2021). Dried mulberry fruit ameliorates cardiovascular and liver histopathological changes in high-fat diet-induced hyperlipidemic mice. *Journal of Traditional and Complementary Medicine*.

[14] Yu Q., Zhao J., Xu Z. (2018). Inulin from Jerusalem artichoke tubers alleviates hyperlipidemia and increases abundance of bifidobacteria in the intestines of hyperlipidemic mice. *Journal of Functional Foods.*.

[15] Zhu Y., Li Q., Mao G. (2014). Optimization of enzyme-assisted extraction and characterization of polysaccharides from *Hericium erinaceus*. *Carbohydrate Polymers*.

[16] Chen H., Zhou X., Zhang J. (2014). Optimization of enzyme assisted extraction of polysaccharides from *Astragalus membranaceus*. *Carbohydrate Polymers*.

[17] Wu J., Yu D., Sun H. (2015). Optimizing the extraction of anti-tumor alkaloids from the stem of *Berberis amurensis* by response surface methodology. *Industrial Crops and Products*.

[18] Zhao C., Li Z., Li C. (2015). Optimized extraction of polysaccharides from *Taxus chinensis* var. *mairei* fruits and its antitumor activity. *International Journal of Biological Macromolecules*.

[19] Zhang L., Guo S., Wang M., He L. (2015). PEG-based ultrasound-assisted enzymatic extraction of polysaccharides from *Ginkgo biloba* leaves. *International Journal of Biological Macromolecules.*.

[20] Wu S., Gong G., Wang Y. (2013). Response surface optimization of enzyme-assisted extraction polysaccharides from *Dictyophora indusiata*. *International Journal of Biological Macromolecules.*.

[21] Zhao Y., Zhang Y., Su P., Yang J., Huang L., Gao W. (2018). Genetic transformation system for woody plant Tripterygium wilfordii and its application to product natural Celastrol. *Frontiers in Plant Science*.

[22] Bevan L., Jones M., Zheng Y. (2021). Optimisation of nitrogen, phosphorus, and potassium for soilless production of Cannabis sativa in the flowering stage using response surface analysis. *Frontiers in Plant Science*.

[23] Wei H., Yue S., Zhang S., Lu L. (2018). Lipid-lowering effect of the Pleurotus eryngii (King Oyster mushroom) polysaccharide from solid-state fermentation on both macrophage-derived foam cells and zebrafish models. *Polymers (Basel).*.

[24] Juneidi S., Gao Z., Yin H. (2020). Breaking the summer dormancy of Pinellia ternata by introducing a heat tolerance receptor-like kinase ERECTA gene. *Frontiers in Plant Science*.

[25] Ren F., Chen Q., Meng C. (2021). Serum metabonomics revealed the mechanism of *Ganoderma amboinense* polysaccharides in preventing non-alcoholic fatty liver disease (NAFLD) induced by high-fat diet. *Journal of Functional Foods*.

[26] Yang H., Xie J., Wang N. (2021). Effects of Miao sour soup on hyperlipidemia in high-fat diet-induced obese rats via the AMPK signaling pathway. *Food Science & Nutrition*.

[27] Wang Q., Mu R. F., Liu X. (2020). Steaming changes the composition of saponins ofPanax notoginseng(Burk.) F.H. Chen that function in treatment of hyperlipidemia and obesity. *Journal of Agricultural and Food Chemistry*.

[28] Cao Y., Zou L., Li W., Song Y., Zhao G., Hu Y. (2020). Dietary quinoa (*Chenopodium quinoa* Willd.) polysaccharides ameliorate high- fat diet-induced hyperlipidemia and modulate gut microbiota. *International Journal of Biological Macromolecules*.

