# Supplementary material for: Extraction of Radix trichosanthis Polysaccharides for Potential Antihyperlipidemic Application
Source: Biomed Res Int. 2022 Apr 11;2022:3811036. doi: 10.1155/2022/3811036 (PMC9015872; doi:10.1155/2022/3811036)
Supplement: Supplementary Materials — Fig. S1: effect of extraction time on the extraction yield of RTPs (pH = 5.5, liquid-to-solid ratio = 30, extraction temperature = 50°C). Supplementary Table S1: one-way analysis of variance (ANOVA) in an orthogonal test. Supplementary Table S2: estimated regression coefficients for the quadratic polynomial model and the analysis of variance for the experimental results [file 3811036.f1.doc]

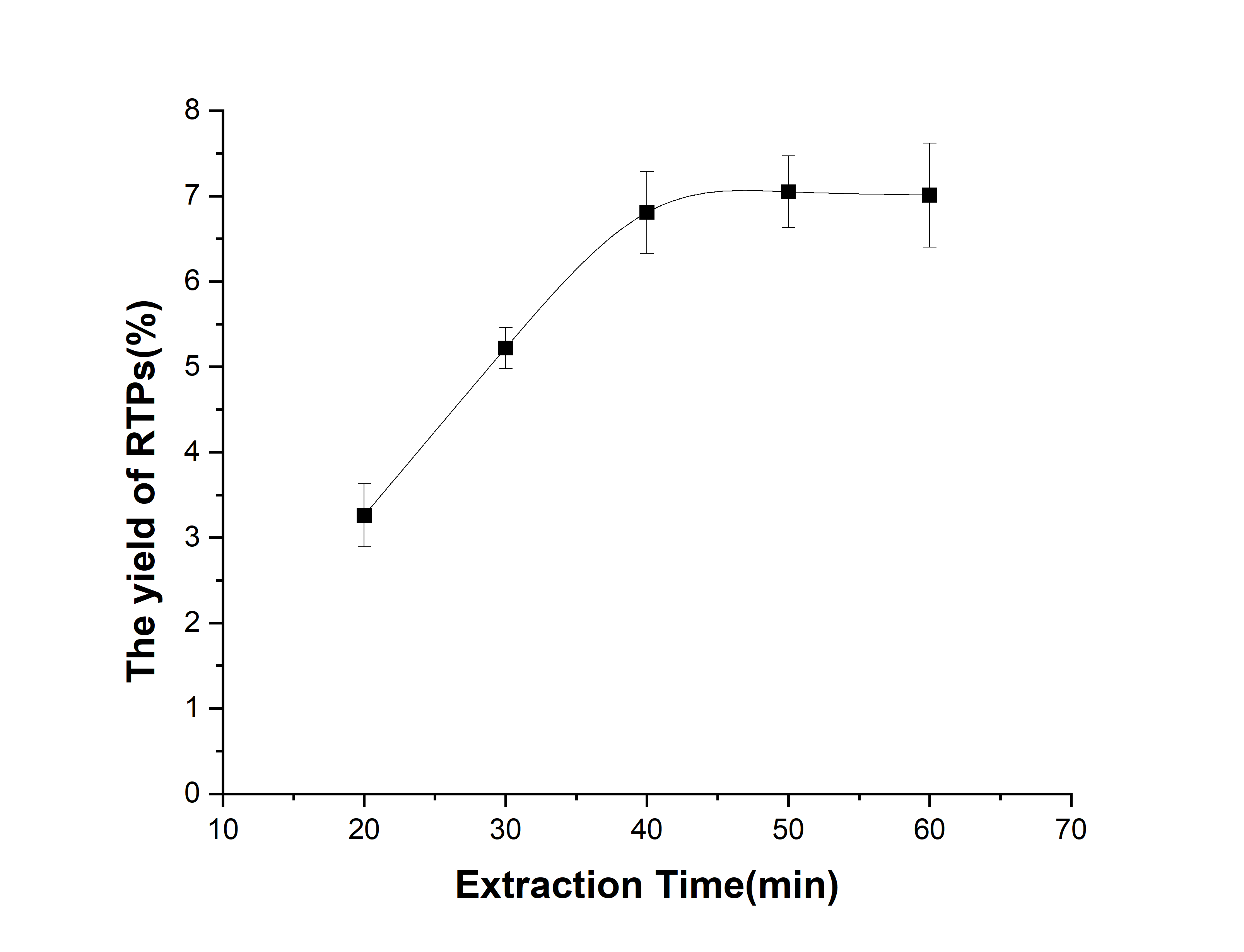


**Fig. S1 Effect of Extraction Time on the extraction yield of RTPs (pH = 5.5, liquid-to-solid ratio = 30, Extraction Temperture = 50 ℃).**

**Supplementary Table S1 Analyses of Variance (ANOVA) in Orthogonal test**

| **Factor** | **Sum of squares** | **Degree of freedom** | **F ratio** | **F critical value** | **Significance** |
| --- | --- | --- | --- | --- | --- |
| **A** | **13.909** | **2** | **19.984** | **19** | ***** |
| **B** | **5.224** | **2** | **7.506** | **19** |  |
| **C** | **0.618** | **2** | **0.888** | **19** |  |
| **error** | **0.7** | **2** |  |  |  |

**A, cellulase, B, papain, C, pectase; **p* < 0.05**

**Supplementary Table S2 Estimated regression coefficients for the quadratic polynomial model and the analysis of variance for the experimental results**

| **Source** | **Sum of squares** | | **Degree of freedom** | | | | **Mean square** | **F value** | | | | ***p* value** | |
| --- | --- | --- | --- | --- | --- | --- | --- | --- | --- | --- | --- | --- | --- |
| **Model** | 63.67 | | | | 9 | | 7.07 | 1518.86 | | | < 0.0001** | | |
| **X_1_** | 32.52 | | | | 1 | | 32.52 | 6982.20 | | | < 0.0001** | | |
| **X_2_** | 2.812×10^-3^ | | | | 1 | | 2.812×10^-3^ | 0.60 | | | 0.4626 | | |
| **X_3_** | 0.74 | | | | 1 | | 0.74 | 159.77 | | | < 0.0001** | | |
| **X_1_X_2_** | 2.5×10^-5^ | | | | 1 | | 2.5×10^-5^ | 5.367×10^-3^ | | | 0.9436 | | |
| **X_1_X_3_** | 0.036 | | | | 1 | | 0.036 | 7.75 | | | 0.0271***** | | |
| **X_2_X_3_** | 4×10^-4^ | | | | 1 | | 4×10^-4^ | 0.086 | | | 0.7780 | | |
| **X_1_^2^** | 26.85 | | | | 1 | | 26.85 | 5764.46 | | | <0.0001** | | |
| **X_2_^2^** | 1.10 | | | | 1 | | 1.10 | 235.35 | | | <0.0001** | | |
| **X_3_^2^** | 0.96 | | | | 1 | | 0.96 | 372.63 | | | <0.0001** | | |
| **Residual** | | 0.033 | | 7 | | | 4.658×10^-3^ | |  |  | | |  |
| **Lack of fit** | | 0.026 | | 3 | | | 8.775×10^-3^ | 5.59 | | 0.0649 not significant | | | |
| **Pure error** | | 6.28×10^-3^ | | 4 | | | 1.57×10^-3^ |  | |
| **Cor total** | | 63.70 | | 16 | | |  | |  |  | | |  |
| **R^2^** | | 0.9995 | |  | | **Adj-R^2^** | | 0.9988 | | | | |  |
| **Pred-R^2^** | | 0.9932 | |  | | **C.V.%** | | 1.23 | | | | |  |

***0.01≤*p*＜0.05**
**** *p*＜0.01**
